# Is the TCA cycle malate dehydrogenase-citrate synthase metabolon an illusion?

**DOI:** 10.1042/EBC20230084

**Published:** 2024-10-03

**Authors:** Joy Omini, Taiwo Dele-Osibanjo, Heejeong Kim, Jing Zhang, Toshihiro Obata

**Affiliations:** 1 Department of Biochemistry, University of Nebraska-Lincoln, Lincoln, NE 68588, U.S.A.; 2 Center for Plant Science Innovation, University of Nebraska-Lincoln, Lincoln, NE 68588, U.S.A.

**Keywords:** citrate synthase, malate dehydrogenase, metabolon, TCA cycle

## Abstract

This review discusses the intriguing yet controversial concept of metabolons, focusing on the malate dehydrogenase-citrate synthase (MDH-CISY) metabolon as a model. Metabolons are multienzyme complexes composed of enzymes that catalyze sequential reactions in metabolic pathways. Metabolons have been proposed to enhance metabolic pathway efficiency by facilitating substrate channeling. However, there is skepticism about the presence of metabolons and their functionality in physiological conditions *in vivo*. We address the skepticism by reviewing compelling evidence supporting the existence of the MDH-CISY metabolon and highlighting its potential functions in cellular metabolism. The electrostatic interaction between MDH and CISY and the intermediate oxaloacetate, channeled within the metabolon, has been demonstrated using various experimental techniques, including protein–protein interaction assays, isotope dilution studies, and enzyme coupling assays. Regardless of the wealth of *in vitro* evidence, further validation is required to elucidate the functionality of MDH-CISY metabolons in living systems using advanced structural and spatial analysis techniques.

## The MDH-CISY metabolon

A ‘metabolon’ is a molecular machinery first described by Paul Srere in 1985 as a ‘supramolecular complex of sequential metabolic enzymes and cellular structural elements’ [1]. Metabolons bring enzyme active sites into close proximity via transient, noncovalent interactions to facilitate metabolic reactions and obviate unfavorable metabolic cross-talk [2,3]. Metabolons have been identified in primary and secondary metabolic pathways in various organisms, including glycolysis, the TCA cycle, *de novo* purine biosynthesis, fatty acid biosynthesis, and biosynthesis of the cyanogenic glycoside dhurrin [4–7]. Metabolons channel the intermediates of pathway reactions within the enzyme complex to offer several advantages, including increasing the local concentration of substrates in the enzyme’s active site, preventing degradation of labile intermediates, protecting shared intermediates from competing reactions, and overcoming thermodynamically unfavorable equilibria [8–10]. The tricarboxylic acid (TCA) cycle metabolon is the first experimentally demonstrated metabolon [11] and, thus far, the most well-characterized metabolon in various organisms [12–14]. The malate dehydrogenase-citrate synthase (MDH-CISY) multienzyme complex makes up the core of the TCA cycle metabolon conserved in all organisms investigated so far [12]. Paul Srere and Laura Halper first reported the complex formation of sequential TCA cycle enzymes between pig heart mitochondrial MDH and CISY in the presence of polyethylene glycol [15].

With a stoichiometry ratio of 2:1, the MDH-CISY multienzyme complex is composed of two MDH dimers and one CISY dimer, as revealed in various organisms across the domains of life [12,14,16,17]. Cross-linking mass spectrometry experiments revealed that MDH and CISY electrostatically interact and form an electropositive path that connects the MDH and CISY active sites [18,19]. This electropositive path allows for the efficient transfer of the negatively charged reaction intermediate, oxaloacetate (OAA), from MDH to CISY active sites by electrostatically retaining OAA on the enzyme surface while preventing its equilibration with the bulk aqueous phase [20]. Site-directed mutagenesis identified positively charged R65 of the porcine mitochondrial CISY located in the electropositive channel as a key residue involved in both MDH-CISY complex formation and OAA channeling [18]. This OAA channeling is expected to enhance the forward reaction of the TCA cycle in multiple ways, and the dynamic MDH-CISY complex can be involved in the complex regulatory mechanisms of the pathway [21,22].

The initial discovery of the MDH-CISY enzyme aggregates and substrate channeling *in vitro* suggested a novel concept in metabolic regulation and impacted the research field. However, the results sparked arguments, casting doubts on the operation of the MDH-CISY metabolon in living cells due to the technical limitations of convincingly investigating a transient structure and its functions *in vivo*. Over 40 years later, arguments and questions about the existence of the metabolon are still discussed. These arguments make the MDH-CISY metabolon seem like an illusion and impede the acceptance of the general metabolon concept by a considerable fraction of the research community. This review seeks to provide tangible evidence to support the existence and functionality of MDH-CISY metabolons and substrate channeling *in vivo*.

## Addressing the skepticism

### Does the MDH-CISY multienzyme complex occur in physiological conditions?

The discovery of the MDH-CISY metabolon started from the results showing protein coaggregation among the TCA cycle enzymes *in vitro* by techniques including protein cross-linking, electrophoresis, and co-sedimentation [11,23,24]. At the initial stages of metabolon studies, there were arguments about the existence of metabolon in physiological conditions *in vivo*. The initial reports of the MDH-CISY metabolon were unconvincing due to the techniques used to demonstrate interaction [25]. One of these is the use of polyethylene glycol as a crowding agent for initial *in vitro* experiments. Although the use of polyethylene glycol is justified to mimic a viscous mitochondrial matrix environment, it also enhances nonspecific macromolecular interactions [10], and extensive attempts to show the existence of the metabolon *in vitro* in the absence of polyethylene glycol failed [26].

Thanks to the emerging techniques for investigating protein–protein interactions, numerous approaches have shown the interaction between mitochondrial MDH and CISY both *in vitro* and *in vivo* in various organisms. Fourier transform surface plasmon resonance (FT-SPR) showed a significant wavenumber shift when mitochondrial MDH and CISY co-exist, indicating the protein–protein interaction between these enzymes [27]. Microscale thermophoresis also clearly detected the interaction between mitochondrial MDH and CISY with a *K*_d_ of 2.29 ± 0.46 µM [22]. *In vivo* interaction between MDH and CISY in *Bacillus subtilis* was demonstrated using a combination of the Strep-protein interaction experiment (SPINE) and Western blot method. The SPINE method co-purified MDH with Strep-tagged CISY followed by *in vivo* cross-linking by formaldehyde for proteins in close proximity (<2 Å) [14]. MDH-CISY interaction was also detected in the mitochondria of a model plant, *Arabidopsis thaliana*, using multiple techniques, including split-luciferase, affinity purification-mass spectrometry assay, yeast two-hybrid, and bimolecular fluorescence complementation [12]. The *in vitro* results were obtained under physiological conditions without an artificial crowding agent, confirming the multienzyme interaction between MDH and CISY, and the *in vivo* results support the protein complex formation in physiological conditions. The consistency of this interaction across different domains of life implies that it may have a significant role in living cells.

### Does substrate channeling occur in the MDH-CISY complex *in vivo*?

Substrate channeling (a.k.a. metabolite channeling) is the core characteristic of a metabolon that distinguishes it from other protein complexes [28]. Due to technical difficulties, there are limited experimental studies supporting substrate channeling in multienzyme complexes [29]. Fortunately, OAA channeling in the MDH-CISY metabolon has been shown in various studies.

**Figure 1:**
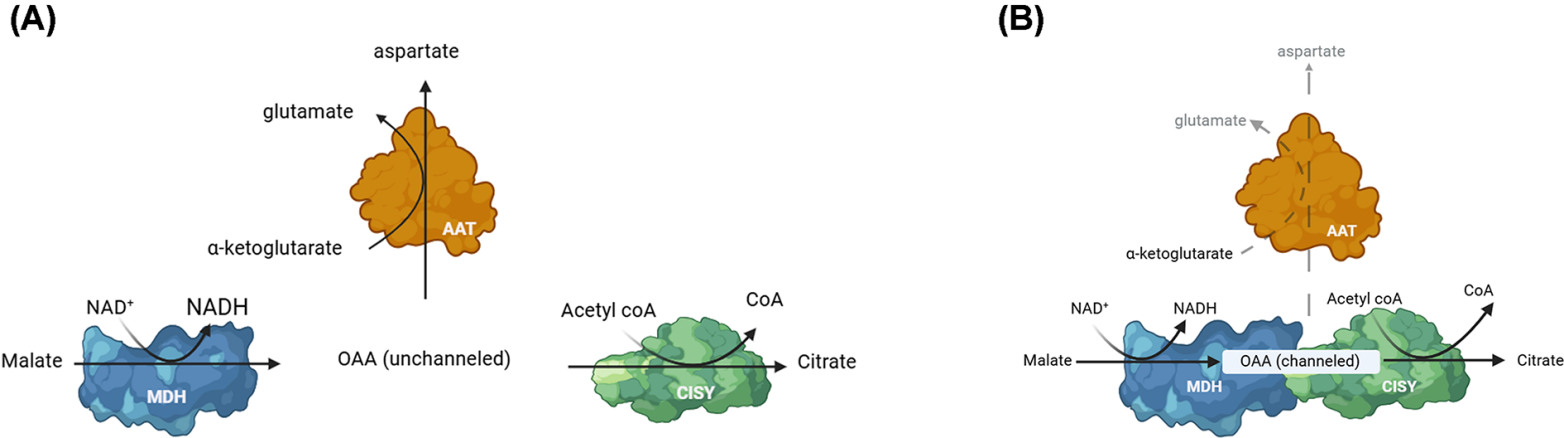
MDH-CISY metabolon protects oxaloacetate (OAA) from competing pathways (**A**) When MDH and CISY do not form a complex, OAA diffusion into the bulk phase allows aspartate aminotransferase (AAT) to compete with CISY for OAA and produce aspartate. (**B**) OAA channeling in the MDH-CISY metabolon protects OAA from AAT.

The isotope dilution experiment is a gold standard to show substrate channeling. In this experiment, an isotopically labeled initial substrate of multistep reactions is supplied, and the isotope accumulation in the final product is monitored following the addition of a large amount of unlabeled intermediate to the system. If the intermediate is channeled, the isotope accumulation in the product will be unaffected by the unlabeled intermediate since the enzyme uses only the labeled intermediate channeled through the metabolon [30]. While isotope dilution experiments have shown substrate channeling in other TCA cycle enzyme complexes *in vitro* and in plant mitochondria [12,31,32], this technique has not been applied for OAA channeling in the MDH-CISY metabolon. This may be partly due to OAA’s instability, especially *in vivo* [33,34]. OAA is spontaneously decarboxylated to pyruvate with a half-life of 14 h at 25°C in a biological aqueous solution (pH 7.4) [33]. Enzymatic decarboxylation enhances the rate of OAA degradation by 2.7 × 10^8^ fold and limits its use for *in vivo* experiments [35,36]. Instead, coupled enzyme reaction assays consisting of aspartate aminotransferase (AAT) and OAA decarboxylase, which compete for OAA, were used to show channeling in the MDH-CISY metabolon *in vitro* [10,18,37]. In these experiments, citrate production from malate was monitored in the presence of MDH and CISY. While the addition of the competing enzymes decreased the citrate production rate by 90% [18,37], AAT could not catalyze the transfer of an amino group to OAA when MDH and CISY were aggregated by polyethylene glycol (Figure 1) [10]. AAT competes less effectively for OAA with an MDH-CISY fusion enzyme compared with free enzymes, indicating that enzyme proximity enhances the channeling and prevents the competing AAT reaction [38]. A transgenic yeast strain expressing this MDH-CISY fusion protein showed a distinctive ^13^C accumulation pattern in the carbon atom positions in alanine, a downstream metabolite derived from OAA, indicating a tight channeling of OAA *in vivo* [17]. These results confirm that the MDH-CISY multienzyme complex is a metabolon to channel OAA. Brownian dynamic simulations detected substrate transfer only in the presence of electrostatic forces, indicating that electrostatic channeling is involved in the transfer of OAA between MDH and CISY [20]. The results also provide insight into the metabolic functions of the MDH-CISY metabolon, specifically in sequestering OAA from competing enzymes.

### Does MDH-CISY metabolon formation enhance pathway reactions?

Another major objection is against the proposed metabolon function of shortening the reaction time by circumventing the intermediate metabolite diffusion time. Diffusion rates of metabolites and enzymes in the cellular matrix are much higher than enzyme turnover rates [39–41]. Therefore, metabolic processes are considered reaction-controlled and not limited by diffusion, and metabolons cannot shorten the reaction time by overcoming diffusion [25,40,42,43].

Metabolic reactions do not occur in an equally distributed bulk aqueous solution in a test tube but rather in a highly crowded cellular matrix with various micro- and macromolecules and a small amount of water. The intracellular matrix shows highly viscous, glass-forming liquid-like characteristics, which will significantly interfere with molecular diffusion [44]. Taking these characteristics into account, the time for metabolite diffusion is estimated to account for 14.4% of the total reaction time of the bacterial glycolytic pathway [40]. The mitochondrial matrix is also crowded and highly viscous [40]. A microscopic measurement suggested that the metabolite and enzyme diffusion rate is three times slower in the mitochondrial matrix than in a saline solution [45], which can theoretically limit TCA cycle flux.

Metabolite diffusion lowers the pathway reaction rates not only by increasing the traveling time between the enzymes but also by diluting the metabolites in cellular bulk phases, exposing them to competing pathways, and causing the degradation of unstable metabolites. These factors apply to the MDH-CISY metabolon, where the channeled intermediate, OAA, is shared with AAT (Figure  1), and OAA is highly unstable and easily degrades in solution [33,34]. The free OAA concentration measured in the mitochondrial matrix is very low, approximately 40 nM [15,46,47]. This low OAA concentration likely limits the CISY reaction [48,49] and is insufficient to achieve the TCA cycle flux rate observed *in vivo* [25,50]. Metabolons and substrate channeling can preserve labile intermediates and increase their local concentration at the enzyme active site [51]. Channeling also helps to reduce the transient time, which is the time required for accumulating intermediates within the enzyme active site to ideal concentrations necessary to maintain flux [52]. A study comparing the initial lag phase of reaction kinetics of the coupled enzyme reaction (reaction generating citrate from malate) by the MDH-CISY fusion protein and the mixture of free enzymes indicated that OAA channeling within the MDH-CISY metabolon reduced the transient time by approximately 2-fold *in vitro* [38,53]. In another *in vitro* study, the transient time of the MDH and wildtype CISY mixture was 30 times shorter than MDH with channeling mutant CISY R65A [18]. Thus, OAA channeling can increase the TCA cycle pathway driving force by increasing the local OAA concentration at the CISY catalytic site, making the observed TCA cycle flux feasible [21]. The MDH-CISY metabolon function can explain the disparity between the apparent OAA concentration and the observed TCA cycle flux.

**Figure 2:**
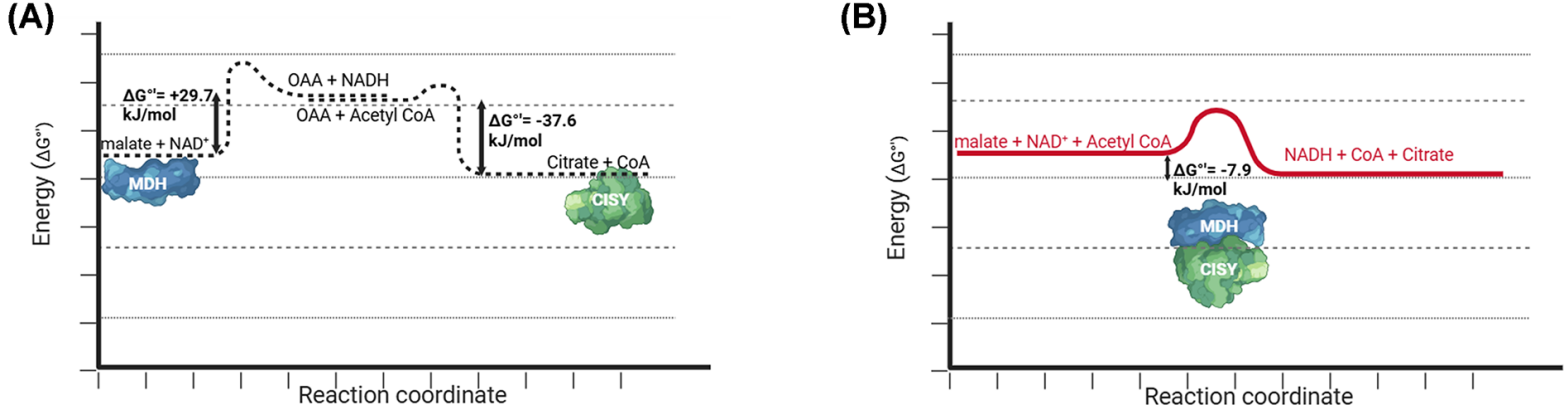
MDH-CISY metabolon couples the endergonic MDH reaction to the exergonic CISY reaction by substrate channeling to yield an overall exergonic reaction (**A**) Reaction profile of the MDH and CISY reactions. The energy absorbed or released during the reactions are shown beside the reaction profile. (**B**) Reaction profile of the MDH-CISY coupled reaction. The energy released as a result of coupling the MDH and CISY reactions is shown beneath the reaction profile.

Additionally, substrate channeling can allow thermodynamically unfavorable reactions to occur. MDH catalyzes a reversible reaction; the forward reaction toward OAA production is thermodynamically unfavorable with a Δ*G*°′ value of +29.7 kJ mol^−1^ [54], while the reverse reaction towards malate production is spontaneous. Substrate channeling allows the coupling of multiple reactions to diminish the energetic limitation associated with the thermodynamically unfavorable one [55]. The MDH-CISY metabolon couples the endergonic MDH reaction to the exergonic citrate synthase reaction, which has a Δ*G*°′ value of −37.6 kJ mol^−1^ [56], yielding a net forward reaction despite thermodynamic barriers (Figure 2). The metabolon transfers OAA directly to the CISY active site, thus keeping it at a low enough concentration within the MDH active site to drive the forward reaction and increase the overall reaction rate. When MDH and CISY reactions were coupled by enzyme immobilization, the coupled MDH-CISY system produced citrate at a faster rate than the free enzymes [46], experimentally indicating the function of the MDH-CISY metabolon.

The significant changes in transient time and reaction rate associated with substrate channeling show that diffusion is likely a limiting factor for the MDH and CISY reactions, which involves a thermodynamically unfavorable reaction and a labile, shared substrate.

### Is MDH-CISY metabolon formation costly for the cell?

Other salient contentions were made about the cellular energy cost for maintaining enzyme complexes. The decrease in entropy upon binding proteins to form a complex is often quite unfavorable and is associated with a positive and non-spontaneous free energy change [57]. Although no energy input is required to maintain a disorderly system with metabolites diffusing freely in the bulk phase, the cell may need to spend energy to maintain organized metabolons [25]. Why will the cell choose an energy-consuming process to channel metabolites over freely diffusing metabolites and enzymes?

Metabolons and substrate channeling can provide an energy-efficient alternative to regulate metabolic pathways, compared with regulation by synthesis and degradation of enzymes [28]. The dynamic nature of metabolons can allow for quick metabolic pathway flux regulation and redirection [7]. The MDH-CISY metabolon is formed by weak electrostatic interactions involving two arginine residues on the CISY surface [10,18]. Weak electrostatic interactions require no energy input but are affected by environmental conditions like pH, ionic strength, and distance between enzymes [58]. Therefore, the MDH-CISY metabolon interaction can be enhanced or disrupted by different factors in the mitochondrial matrix, including pH, redox state, and metabolite abundance (Table 1), which reflect cellular metabolic status. *In vitro* biophysical protein interaction assays using microscale thermophoresis showed that substrates of the MDH and CISY reactions like malate, NAD^+^, and acetyl-CoA enhanced the association of the MDH-CISY metabolon while products like citrate and NADH decreased it [22]. Results of computational modeling showed that allosteric effectors such as acetyl CoA possibly modulate MDH-CISY interaction by modifying CISY conformation [22]. Succinate, α-ketoglutarate, and a slightly acidic pH condition also enhanced the interaction of the metabolon [22]. Similarly, fluorescence anisotropy assays showed that NADH decreased the MDH-CISY interaction while α-ketoglutarate increased it [13]. A microfluidic channel assay revealed that an increase in malate concentration in the vicinity of the MDH and CISY enzymes induces chemotactic motion, bringing the enzymes together and enhancing the co-diffusion of mitochondrial MDH and CISY [16]. The MDH-CISY co-diffusion is inhibited by ATP, which inhibits OAA generation [16]. These effects on the MDH-CISY metabolon formation are likely due to allosteric changes in protein conformations and can offer feedback regulations of pathway reactions [22]. Other post-translational modifications, such as acetylation and phosphorylation, also influence metabolon formation [59,60], although these are yet to be explored in the TCA cycle MDH-CISY metabolon studies.

**Table 1:** Effects of different factors on the MDH-CISY protein interaction

Factors	Effects on MDH-CISY interaction	Methods	References
Malate	Enhance	MST, microfluidic channel	[16,22]
NAD^+^	Enhance	MST, microfluidic channel	[16,22]
NADH	Disrupt	MST, fluorescence anisotropy	[13,22]
ATP 25 mM	Disrupt	Microfluidic channel	[16,22]
10 mM	Enhance	MST	
Acetyl CoA	Enhance	MST, microfluidic channel	[16,22]
α-Ketoglutarate	Enhance	MST, fluorescence anisotropy	[13,22]
Succinate	Enhance	MST	[22]
Acidic pH	Enhance	MST	[22]
Citrate	Disrupt	MST	[22]
Nitric oxide	Enhance	Fourier transform surface plasmon resonance	[25]

Abbreviation: MST, microscale thermophoresis.

These *in vitro* MDH-CISY interaction studies were conducted with no external energy input, showing that the MDH-CISY metabolon requires little or no energy input to maintain and regulate the interaction. The dynamic MDH-CISY metabolon can offer energy-efficient fine-tuning of the TCA cycle and adjacent pathways by autonomous control of multienzyme complex formation in response to the physicochemical microenvironment in the mitochondrial matrix associated with cellular metabolic demands.

Evidence indicates the MDH-CISY metabolon is not an illusion, but some critical facts remain to be shown. Here, we have addressed skepticism about the MDH-CISY metabolon and provided evidence supporting its existence *in vivo*. We have also discussed the possible functions of the MDH-CISY metabolon, highlighting its role in supporting the high metabolic flux through the TCA cycle in living cells. The cumulative evidence indicates the existence of a functional MDH-CISY metabolon. Thus, we would like to state here that the MDH-CISY metabolon is NOT an illusion.

However, theoretical advantages and functions proposed so far are still largely based on the *in vitro* evidence, and experimental *in vivo* validation is limited because of technological restrictions. The MDH-CISY multienzyme complex is likely a part of the larger TCA cycle metabolon involving other pathway enzymes [11,12]. However, the fragile nature of metabolons makes it challenging to isolate or reconstruct the intact TCA cycle metabolon for *in vitro* investigation of functions [10,16]. It also prevents identifying the components of the intact TCA cycle metabolon and analyzing their dynamics *in vivo* [12]. So far, the technical challenges to analyze a large, fragile protein complex limit our understanding of the roles of the MDH-CISY complex in a larger TCA cycle metabolon.

This review outlined the advantages of the MDH-CISY metabolon and OAA channeling, such as protecting intermediates from competing pathways, increasing the local concentration of intermediate in the enzyme active site, and reducing reaction transient time. These functions need to be studied in living systems to elucidate the functions of the MDH-CISY metabolon in metabolic pathway regulation. Dynamic association and dissociation of the MDH-CISY metabolon can offer energy-efficient regulation of the TCA cycle and branching pathways, but neither the dynamic metabolon nor its effects on metabolic pathway fluxes have been experimentally demonstrated in living systems.

Currently, there are promising methods that can be employed to study the role of metabolons in metabolic flux regulation *in vivo*. Stable isotope tracer analyses, including steady-state and isotopic nonstationary metabolic flux analyses, can be used to determine *in vivo* metabolic pathway flux. These analyses take advantage of the isotope label enrichment and the signature in the labeling patterns of metabolites produced by different pathways to detect the signs of substrate channeling [29]. Advances in structural biological techniques like cryo-electron microscopy and tomography [61], spatial metabolomics technologies [62], and real-time *in vivo* protein interaction assays [63,64] will facilitate the study of enzyme and metabolite localization to investigate metabolons in action. We are confident that with careful investigations using these methods, the functionality of the MDH-CISY metabolon will be revealed to facilitate our understanding of metabolic pathway regulation in living cells by showing a metabolon as a novel regulatory layer.

## Summary

- A ‘metabolon’ is a dynamic multienzyme complex that mediates substrate channeling and is potentially involved in metabolic pathway regulation, but skepticism about its existence and functionality has hindered the acceptance of this concept by a significant fraction of the research community.
- The MDH-CISY metabolon can offer many advantages to maintain high metabolic flux through the tricarboxylic acid cycle, which may not be possible without oxaloacetate channeling through the MDH-CISY metabolon.
- The MDH-CISY multienzyme complex dynamics, its roles in the large TCA cycle metabolons, and influence on metabolic pathway flux should be evaluated in living systems to fully prove its functionality and roles in metabolic network regulation.

## Abbreviations

AAT: aspartate aminotransferase
BIFC: bimolecular fluorescence complementation
CISY: citrate synthase
FT-SPR: Fourier transform surface plasmon resonance
*K*_d_: dissociation constant
MDH: malate dehydrogenase
MST: microscale thermophoresis
OAA: oxaloacetate
pI: isoelectric point
SPINE: Strep-protein interaction experiment
TCA: tricarboxylic acid
